# Subtractive CRISPR screen identifies the ATG16L1/vacuolar ATPase axis as required for non-canonical LC3 lipidation

**DOI:** 10.1016/j.celrep.2021.109899

**Published:** 2021-10-26

**Authors:** Rachel Ulferts, Elena Marcassa, Lewis Timimi, Liam Changwoo Lee, Andrew Daley, Beatriz Montaner, Suzanne Dawn Turner, Oliver Florey, John Kenneth Baillie, Rupert Beale

**Affiliations:** 1 The Francis Crick Institute, London, UK; 2 Department of Pathology, University of Cambridge, Cambridge, UK; 3 Signalling Programme, Babraham Institute, Cambridge, UK; 4 University of Edinburgh, Edinburgh, UK; 5 Division of Medicine, UCL, London, UK

**Keywords:** autophagy, influenza, ATG16L1, v-ATPase, ATG4D, RALGAP

## Abstract

Although commonly associated with autophagosomes, LC3 can also be recruited to membranes by covalent lipidation in a variety of non-canonical contexts. These include responses to ionophores such as the M2 proton channel of influenza A virus. We report a subtractive CRISPR screen that identifies factors required for non-canonical LC3 lipidation. As well as the enzyme complexes directly responsible for LC3 lipidation in all contexts, we show the RALGAP complex is important for M2-induced, but not ionophore drug-induced, LC3 lipidation. In contrast, ATG4D is responsible for LC3 recycling in M2-induced and basal LC3 lipidation. Identification of a vacuolar ATPase subunit in the screen suggests a common mechanism for non-canonical LC3 recruitment. Influenza-induced and ionophore drug-induced LC3 lipidation lead to association of the vacuolar ATPase and ATG16L1 and can be antagonized by *Salmonella* SopF. LC3 recruitment to erroneously neutral compartments may therefore represent a response to damage caused by diverse invasive pathogens.

## Highlights

• Subtractive CRISPR screen identifies genes involved in non-canonical LC3 lipidation
• v-ATPase regulates LC3 lipidation at erroneously neutral compartments
• RALGAP complex involved in M2 proton channel induced LC3 lipidation
• ATG4D is responsible for LC3 recycling in M2-induced and basal LC3 lipidation

## Graphical abstract

**Figure:**
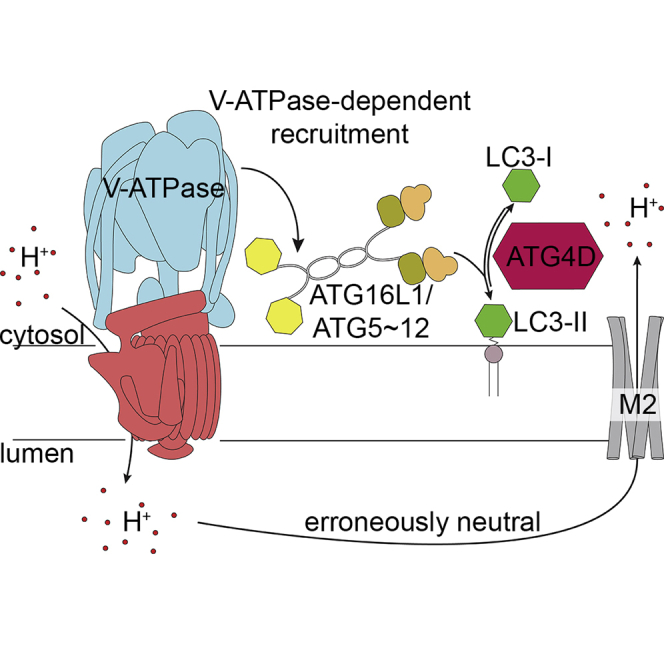

## Teaser

Ulferts et al. identify v-ATPase as the central regulator of ATG16L1 WD40 domain-dependent of LC3 lipidation. This lipidation can be prevented by *Salmonella* SopF and counteracted by ATG4D, the predominant ATG4 paralog responsible for LC3 delipidation. The RalGAP complex affects influenza virus M2-induced LC3 lipidation by affecting M2 trafficking.

## Introduction

Autophagy is a catabolic process characterized by the delivery of cytoplasmic material to the lysosome for degradation (Mizushima and Komatsu, 2011). A key feature of this pathway is the relocalization of microtubule-associated proteins 1A/1B light chain 3 (LC3). Upon induction of autophagy, LC3 becomes covalently conjugated to phosphatidylethanolamine (PE) at sites forming double-membrane autophagosomes (Mizushima et al., 1998). Lipidation depends on the activity of two ubiquitin-like conjugation systems comprising ATG3, ATG5, ATG7, ATG10, and ATG12 (Kaufmann et al., 2014). ATG5 and ATG12 form a complex with ATG16L1 that catalyzes the transfer of activated LC3 to PE, in a manner analogous to an E3 ligase. Localization of this complex determines site specificity of LC3 lipidation (Fujioka et al., 2014). The soluble form of LC3 is referred to as LC3-I, and the PE-conjugated form as LC3-II.

LC3-II also decorates various single-membrane compartments in response to different stimuli (Florey et al., 2011; Jacquin et al., 2017; Sanjuan et al., 2007). Examples of this non-canonical pathway include LC3-associated phagocytosis (LAP), micropinocytosis, and entosis (Florey et al., 2011, 2015). In all of these examples a subset of these endocytic vesicles acquires LC3-II. This “non-canonical autophagy” has also been implicated in the regulation of host homeostasis following infection with influenza A virus (IAV) (Fletcher et al., 2018). Upon infection of the cell, the viral M2 protein, a small proton selective ion channel or “viroporin,” promotes LC3 lipidation (Beale et al., 2014; Gannagé et al., 2009; Ren et al., 2015; Zhirnov and Klenk, 2013). LC3-II accumulates at intracellular vesicles and the plasma membrane (Beale et al., 2014). Influenza M2 dissipates intracellular proton gradients, resulting in erroneously neutral compartments (Ciampor et al., 1992; Henkel et al., 1999). LC3 lipidation is dependent on the ion channel activity of M2 (Fletcher et al., 2018; Ren et al., 2015). Additionally, the C-terminal region of the IAV-M2 interacts directly with LC3 through a highly conserved LC3-interacting region (LIR) (Beale et al., 2014). We have recently shown that the molecular mechanism of LC3 lipidation induced by M2 differs from that of canonical, starvation-induced LC3 lipidation. In contrast to canonical autophagy, the recruitment of the E3-like ATG12-ATG5/ATG16L1 complex during M2-induced LC3 lipidation depends on the WD repeat-containing C-terminal domain (WD40 CTD) of ATG16L1 but is independent of FIP200 and WIPI2b binding (Fletcher et al., 2018). The WD40 CTD dependency of ATG16L1 recruitment is also observed during other non-canonical LC3 lipidation events, including responses to ionophores, LAP, and entosis (Fletcher et al., 2018). The molecular mechanism of ATG16L1 recruitment to these membranes is unknown, but provocatively WD40 CTD has been reported to interact with the vacuolar ATPase (v-ATPase) in the context of *Salmonella* infection. This process is antagonized by the *Salmonella* effector SopF (Xu et al., 2019).

To identify the genes involved in this novel cellular pathway, we performed a genome-wide genetic knockout (KO) screen using CRISPR-Cas9 technology. We exploited the resistance of GFP-tagged LC3-II to removal from permeabilized cells by detergents such as saponin to enable us to sort cells based on fluorescence (Eng et al., 2010). To discriminate between true hits and genes that affect expression of the GFP-LC3 marker, we also performed the screen without permeabilization and subtracted these results on a guide-by-guide basis. This differential screen uncovered several host factors involved in the regulation of M2-dependent LC3 lipidation, including all six components of the core lipidation machinery. We also identified a subunit of the v-ATPase (V0A1) and the GTPase activator RALGAP β subunit as required for optimal LC3 lipidation. Conversely, deletion of the cysteine protease ATG4D enhanced LC3 lipidation and accumulation in cells expressing IAV M2.

## Results

### The proton channel activity of M2 is required for M2-induced LC3 lipidation

We previously showed that IAV induces LC3 lipidation and relocalization to the plasma membrane through a pathway that differs from canonical autophagy (Beale et al., 2014). This event is dependent on the proton channel activity of the M2 protein and WD40 CTD of ATG16L1 (Fletcher et al., 2018).

**Figure 1:**
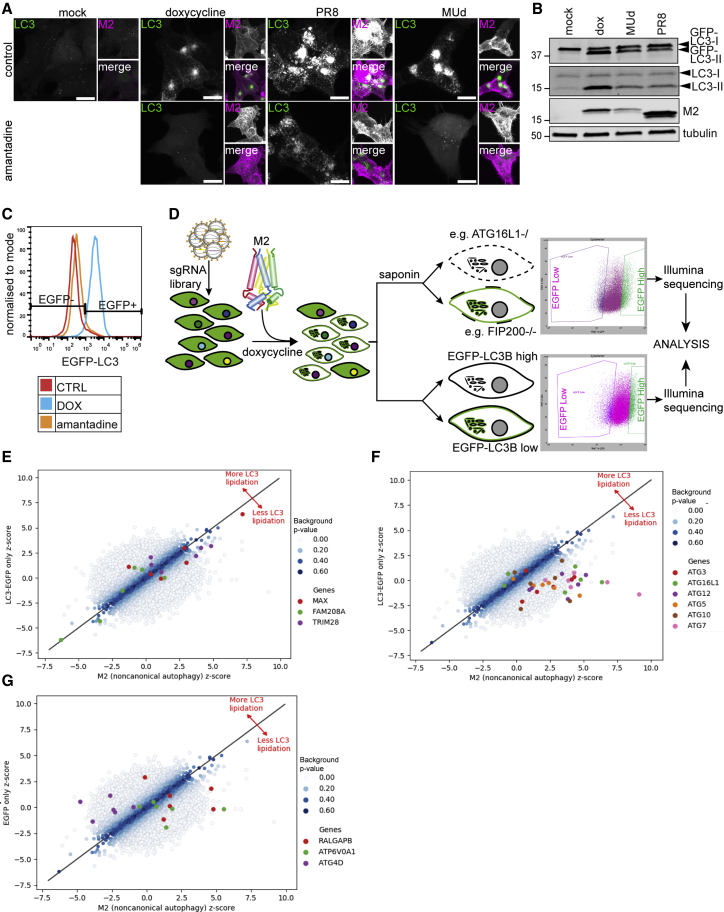
M2 proton channel activity is sufficient for LC3B lipidation (A) Tet-ON M2 cells treated for 16 h with 3 μg/mL doxycycline (dox) or infected with PR8 or MUd at an MOI of 3 plaque-forming units (PFU) per cell, with or without amantadine. Cells were then fixed and stained for M2 protein. Scale bars, 10 μM. (B) Western blot (WB) analysis of LC3 lipidation in HCT116 EFGP-LC3B Tet-ON M2 cells treated for 16 h with dox or infected with PR8 or MUd at an MOI of 3 PFU per cell. (C) FACS analysis of membrane-associated EGFP-LC3B. HCT116 EFGP-LC3B Tet-ON M2 cells were treated for 16 h with 10 μg/mL dox, with or without 5 μM amantadine. (D) Schematic depiction of the CRISPR screen. M2 expression was induced in library-transduced cells, and the top and bottom 10% of EGFP-expressing cells were sorted into permeabilized M2-expressing cells (top) and in unpermeabilized cells (bottom). Factors involved in M2-induced LC3 lipidation were identified by subtractive comparison of permeabilized and unpermeabilized treatment conditions. (E–G) Scatterplots showing the representation of sgRNAs (E) affecting EGFP-LC3B expression, (F) targeting the core LC3 lipidation machinery, and (G) targeting selected genes required for or counteracting LC3 lipidation.

Other WD40 CTD-dependent LC3 lipidation processes target endo-lysosomal vesicles and thus components overlap with the pathway required for IAV entry into the cell (Florey et al., 2011; Sanjuan et al., 2007). Overlapping requirements for M2-induced LC3 lipidation and virus entry might therefore obscure target hits in a screen using virus infection. Alternatively, ectopic expression of IAV M2 has been shown to be sufficient for the induction of proton channel-dependent LC3 lipidation in the absence of other viral components (Ren et al., 2015; Zhirnov and Klenk, 2013). We therefore established a doxycycline-inducible M2-expression cell line. We chose the M2 protein of IAV strain A/Udorn/72, as its proton channel is sensitive to inhibition by amantadine. Expression of M2 led to LC3 relocalization and lipidation similar to that observed after infection with IAV (Figures 1A and 1B). Relocalization was inhibited by amantadine in M2-expressing and MUd (a reassortant strain of IAV A/Rico/8/34 [PR8] with the M2-encoding segment of strain Udorn)-infected cells but not in cells infected with the amantadine-resistant strain PR8, confirming that the proton channel activity of M2 is required for M2-induced LC3 lipidation (Figures 1A and 1C).

### A genome-wide CRISPR-Cas9 screen identified factors involved in M2-induced LC3 lipidation

**Figure 2:**
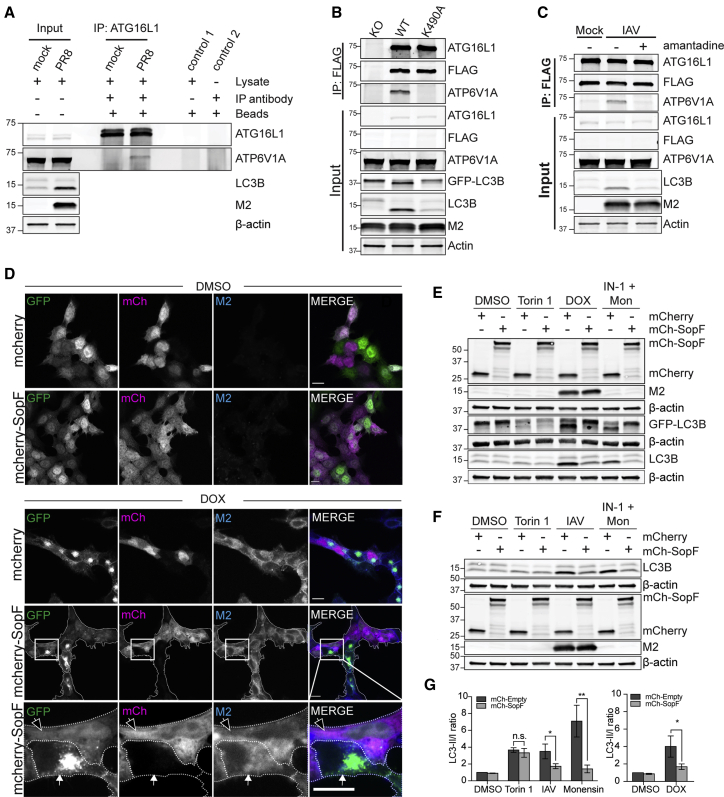
v-ATPase is required for M2-induced LC3 lipidation (A) Immunoprecipitation (IP) analysis of endogenous ATG16L1 in HCT116 cells infected for 16 h with PR8. Control 1, lysate from PR8-infected cells incubated with beads to control for unspecific binding; control 2, beads and ATG16L1 antibody. (B) The M2-induced interaction between ATG16L1 and the v-ATPase depends on K490 of the ATG16L1 CTD. HCT116 ATG16L1^−/−^ cells reconstituted with WT or K490A mutant FLAG-muATG16L1 were infected with PR8 for 16 h followed by IP with anti-FLAG antibody. (C) Induction of ATG16L1-v-ATPase interaction by M2 depends on M2 ion channel activity. HCT116 ATG16L1^−/−^ FLAG-muATG16L1 reconstituted cells were infected with MUd for 16 h. Amantadine was added at 3 days p.i. IP as in (B). (D) Tet-ON M2 cells stably expressing mCherry or mCherry-SopF following treatment with dox or mock treated. M2 expression was detected using the M2-specific antibody 14C2. Black arrow indicates mCherry-SopF-expressing cell; white arrow indicates mCherry-SopF-negative cell. (E) LC3B lipidation analysis in Tet-ON M2 cells stably expressing either mCherry or mCherry-SopF after treatment with either Torin 1 (250 nM for 3 h), dox, or VPS34 IN-1 pretreatment (1 μM for 30 min) followed by monensin (100 μM for 1 h). (F) HCT116 stably expressing mCherry or mCherry-SopF treated as in (E) or infected with PR8 for 16 h (MOI of 10 PFU per cell). (G) Quantification of (E) (right panel) and (F) (left panel). The graph shows fold change in the LC3II/LC3I ratio relative to mCherry DMSO. Bars show mean ± SD. ^∗^p < 0.05, ^∗∗^p < 0.01.

To identify factors involved in this pathway, we performed a CRISPR-Cas9 screen in HCT116 EGFP-LC3 cells expressing the IAV-M2 protein under a doxycycline inducible promoter. Briefly, cells were transduced with the GeCKO library v2 (Sanjana et al., 2014) encoding six guide RNAs (gRNAs) per gene. Cells were expanded for 10 days post-transduction to maximize gene editing. M2 expression was induced for 16 h with doxycycline. Permeabilization of cells with saponin was used to wash away unlipidated LC3 (Eng et al., 2010). Since EGFP fluorescence is the readout, genes involved in EGFP expression from the Moloney murine leukemia virus (MMLV)-based promoter as well as genes involved in LC3 lipidation in this context would be expected to be identified using this method. The pool of cells was therefore split into two, either permeabilized with saponin or not, stained for M2 expression, and sorted by fluorescence-activated cell sorting (FACS) according to the top and bottom 10% EGFP fluorescence levels (Figure 1D). After purification of genomic DNA and sequencing, analysis was performed as per Li et al. (2020). Genes directly responsible for LC3 lipidation were identified in the saponin permeabilized set. Genes such as FAM208A, a component of the HUSH complex, and MAX (MYC associated factor X), a known transcription factor, were identified in both the permeabilized and unpermeabilized screens (Figure 2B; Figure S1). This is expected since the MMLV promotor, which drives GFP-LC3 marker expression, can be silenced by the HUSH complex (Tchasovnikarova et al., 2015). In a z-z scatterplot they therefore align along the z = y axis (Figure 1E). To isolate the genes responsible for promoting or antagonizing non-canonical autophagy, we subtracted the *Z* scores of each guide in the non-permeabilized dataset from the corresponding guides in the permeabilized dataset (Figure S1). This showed the top six hits as required for LC3 lipidation to be ATG7, ATG12, ATG16L1, ATG5, ATG10, and ATG3 (Figure 1F; Figure S1A). This subtractive screening approach therefore correctly identified all components of the core LC3 lipidation machinery. Two other interesting candidate genes were ATP6V0A1 and RALGAPB (Figure 2D), both of which were analyzed further. Only one gene, ATG4D, was strongly identified as a potential antagonist of LC3 lipidation (Figure 1G).

### The role of the v-ATPase in M2-induced LC3 lipidation

v-ATPase has been previously implicated in different types of non-canonical autophagy, including entosis, LAP, and ionophore-induced LC3 lipidation, on the basis of their sensitivity to the v-ATPase inhibitor bafilomycin A1 (Fletcher et al., 2018; Florey et al., 2015). Recent findings have revealed a requirement for the interaction of the v-ATPase complex with ATG16L1 in the selective degradation of bacteria (xenophagy) (Xu et al., 2019). In the same study, the authors demonstrated the ability of the *Salmonella* effector SopF to interfere with this interaction. In light of this new evidence, we sought to investigate a role for this v-ATPase/ATG16L1 axis in the regulation of non-canonical autophagy, including M2-induced lipidation. In common with Xu et al., we failed to obtain KO cell lines, consistent with v-ATPase function being required for cell viability. Following infection with IAV strain PR8, the V1A subunit of the v-ATPase co-immunoprecipitated with endogenous ATG16L1 (Figure 2A). The ionophore drug monensin increases levels of LC3-II by both canonical autophagy and WD40 CTD-dependent LC3 lipidation (Fletcher et al., 2018). By inhibiting canonical autophagy with a VPS34 inhibitor, IN-1, we were able to demonstrate the association of ATG16L1 and the v-ATPase in another WD40 CTD-dependent LC3 lipidation context (Figure S2A). This interaction was further confirmed using a panel of different cell lines after treatment with IN-1/monensin (Figures S2B and 2C). We have previously shown that M2- and ionophore-induced LC3 lipidation depends on a conserved pocket of the WD40 CTD, which includes K490 (Fletcher et al., 2018). To confirm that this is important for v-ATPase-ATG16L1 interaction, we reconstituted HCT116 ATG16L1^−/−^ cells with FLAG-tagged ATG16L1 wild-type (WT) or K490A mutant and tested for v-ATPase interaction in PR8 infection. While an interaction was observed in WT reconstituted cells, substitution K490A abolished this interaction (Figure 2B). Furthermore, the interaction depends on proton gradient dissipation by the M2 ion channel, as this was severely reduced when the ion channel was inhibited with amantadine (Figure 2C). Next, we investigated the effect of expression of the bacterial effector SopF on WD40 CTD domain-dependent LC3 lipidation. This effector inhibits WD40 CTD-dependent LC3 lipidation of the *Salmonella*-containing phagophore (Xu et al., 2019). We generated HCT116-EGFP-LC3B Tet-ON M2 cell lines stably expressing mCherry-SopF or mCherry control. In this system, we were able to compare relocalization after M2 expression or canonical autophagy induction using the mTOR inhibitor, Torin 1. In cells treated with doxycycline, SopF expression inhibited LC3 puncta formation and relocalization to the plasma membrane (Figure 2D). SopF had no effect on Torin 1-induced LC3 puncta formation (Figure S2D), indicating that the inhibition is specific for non-canonical LC3 lipidation. We confirmed this result by western blot (Figure 2E) and in the context of IAV infection (Figures 2F and 2G). Collectively, these data confirm the role of the v-ATPase/ATG16L1 axis in the activation of WD40 CTD-dependent LC3 lipidation.

### The RalGAP complex is important for M2-induced LC3 lipidation

RalGAPβ is the non-catalytic subunit of the Ral GTPase activating protein (RalGAP) complex and forms a heterodimer with a RalGAPα subunit (Shirakawa et al., 2009). This complex acts as a GTPase activator for Ras-like small GTPases RALA and RALB.

**Figure 3:**
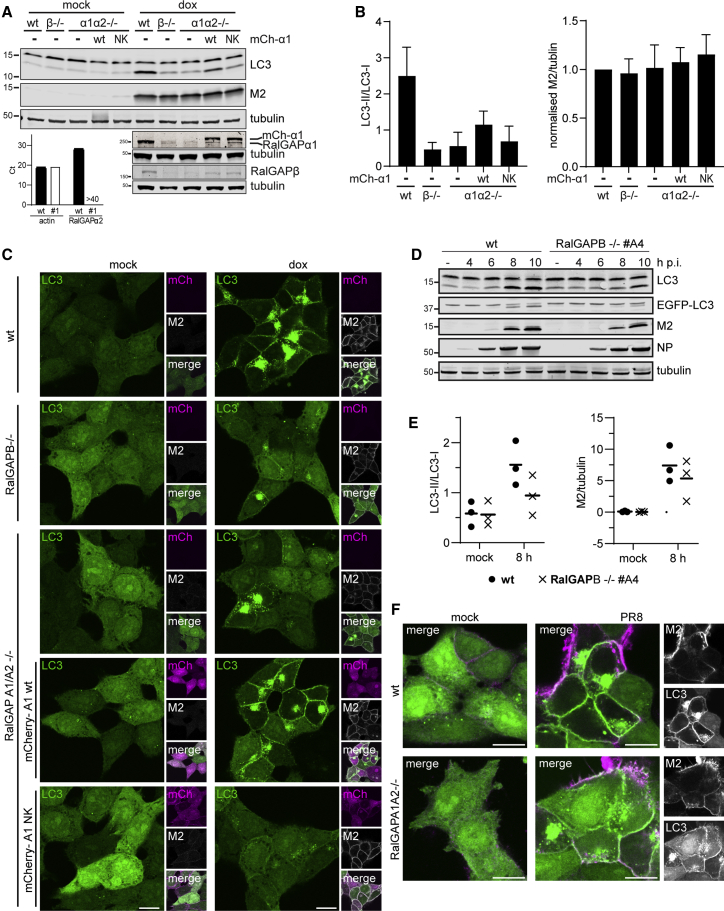
RalGAP depletion inhibits LC3B relocation in response to M2 (A) LC3 lipidation analysis of Tet-ON M2 WT, RalGAPβ^−/−^ (#B8), and RalGAPα1α2^−/−^ (#1) CRISPR knockout (KO) single-cell clones after induction by dox. CRISPR KO (−/−) for RalGAPβ and α1 was analyzed by WB. Ablation of RalGAPα2 expression in RalGAPα1α2^−/−^ #1 was confirmed by qPCR (bar chart inset). Expression of RalGAPα1 WT or N1903K mutant (NK) in RalGAPα1α2^−/−^ cells was reinstated by transduction with mCherry-RalGAP-expressing lentivirus followed by FACS for mCherry expression. (B) Quantification of LC3-II/LC3-I ratio (left panel) and normalized M2 expression (right panel) of (A). Mean ± SD. (C) Immunofluorescence analysis of LC3 relocalization of cells treated as in (A) fixed and stained for M2 protein. Scale bars, 10 μM. (D) LC3 lipidation analysis of Tet-ON-M2 WT or RalGAPβ (#A4) cells after infection with PR8 (MOI of 10 PFU per cell). (E) Quantification of (D). Graphs show mean of LC3II/LC3I ratio (left) and M2 normalized to tubulin (right). (F) Tet-ON-M2 WT and RalGAPA1A2^−/−^ cells infected with PR8 for 8 h, fixed, and stained for M2 protein. Scale bars, 10 μM.

To validate RalGAPβ involvement in the regulation of LC3 lipidation, we generated HCT116 Tet-ON M2 RalGAP KO cells using CRISPR-Cas9 and confirmed the absence of RalGAPβ expression by western blot (Figure 3A). Depletion of RalGAPβ strongly reduced M2-induced LC3 lipidation (Figure 3A) and relocalization of EGFP-LC3B (Figure 3C).

We also analyzed LC3 behavior in cells depleted of the other subunit of the complex, RalGAPα. Mammalian cells encode two paralogs, RalGAPα1 and RalGAPα2. In human cells, these possess 53% identity across the whole sequence and 83% sequence identity in the GAP domain (Martin et al., 2014).

We generated cell clones devoid of expression of both paralogs. Absence of RalGAPα1 expression was confirmed by western blot (Figure 3A), while absence of RalGAPα2 expression, due to the lack of a suitable antiserum, was confirmed by qPCR (Figure 3A, inset). Lack of the RalGAPα subunits also strongly reduced M2-induced EGFP-LC3B relocalization (Figure 3C) and LC3 lipidation (Figures 3A and 3B), implicating the RalGAP complex in M2-induced LC3 lipidation. Neither RalGAPβ nor RalGAPα1/α2 KO affected M2 expression levels (Figures 3A and 3B, right panel).

To further exclude that the observed effects were due to off-target effects of the chosen single guide RNAs (sgRNAs), we expressed RalGAPα1 in RalGAPα1α2^−/−^ cells using lentiviral transduction. The expression level of mCherry-RalGAPα1 (in this polyclonal population) was similar to WT cells (Figure 3A). Partial recovery of M2-induced LC3 lipidation and relocalization was observed (Figures 3B and 3C). Reconstitution of RalGAPβ or RalGAPα2 was not tested, as no stable expression plasmid could be obtained. Expression of the GAP active site mutant, N1093K, did not result in recovery of LC3 lipidation in M2 expression. We observed that ablation of expression of either complex subunit decreased expression levels of the other subunit (Figure 3A). Levels of RalGAPβ recovered upon reexpression of RalGAPα1 WT or N1903K mutant, supporting the successful reconstitution of complex formation (Figure 3A). While there was no effect on M2 expression levels, ablation of RalGAP expression resulted in a reduction of intracellular M2 staining in most cells (Figure 3C; see Figure S3A for larger image). Intracellular localization of M2 could be observed in RalGAPα1 WT reconstituted cells. Interestingly, RalA/B-GTP has been shown to inhibit endocytosis and enhance exocytosis (Moskalenko et al., 2002). Intracellular M2 accumulation was observed in ATG16L1^−/−^ cells where LC3 lipidation is ablated (Figure S3B), indicating that LC3 lipidation of M2-containing vesicles has no gross effect on intracellular M2 localization.

We then tested the effect of RalGAP KO in LC3 lipidation during IAV infection. RalGAPβ^−/−^ cells clone A4 and WT cells were infected with IAV strain PR8 and LC3 lipidation at different time points post-infection (p.i.) analyzed by western blot. While we observed a reduction in LC3 lipidation in RalGAPβ^−/−^ cells, this effect was reduced compared to the M2 expression system (Figures 3D and 3F). We also observed a slight reduction in M2 expression levels (Figure 3E). Identical effects on LC3 lipidation concurrent with a slight reduction in M2 expression levels were observed in infection of RalGAPβ^−/−^ clone B8—which was generated using a different sgRNA to clone A4—or the RalGAPα1α2^−/−^ cell line (Figure S4). No substantial effect on virus replication was found (Figure S4C). The observed effect was thus specific for the depletion of the RalGAP complex, and not a clonal artifact.

The M2 protein used in the expression system was derived from the IAV strain Udorn. However, a similar reduction in LC3 lipidation and M2 expression was observed using the chimeric influenza virus MUd (Figure S4B). This indicates that the effect on LC3 lipidation and M2 expression are not due to strain-specific differences in the M2 protein sequence.

We have previously shown that the dependence of M2-induced LC3 lipidation on the WD40 CTD of ATG16L1 is shared by other non-canonical pathways, namely LAP, entosis, and drug-induced endosomal perturbation (Fletcher et al., 2018). Entosis is a process observed in cancer where cells become entirely engulfed by another cell. Upon death of the internalized cells, the surrounding vesicle membrane of the engulfing cell becomes decorated with LC3 (Florey et al., 2011). To test whether RalGAP is important in LC3 lipidation in this process we analyzed entotic events in RalGAP^−/−^ and WT cells. No significant difference could be observed, indicating that RalGAP is dispensable for this activity (Figure S5A). We also could not observe an effect of RalGAP KO on LC3 lipidation and EGFP-LC3B punctae formation during monensin-induced endosomal perturbation (Figures S5B and S5C).

In summary, we conclude that the RalGAP complex is important for M2-induced LC3 lipidation but does not play a major role in other forms of WD40 CTD domain-dependent LC3 lipidation.

### ATG4D depletion enhances LC3B lipidation levels

ATG4D was the only gene targeted that appeared to give rise to higher LC3 lipidation in the screen. The ATG4 family comprises four paralogs, ATG4A, ATG4B, ATG4C, and ATG4D. During autophagy, ATG4 is required both for the processing of the newly synthesized pro-LC3/GABARAP and for the recycling of the PE-conjugated from the autophagosomal membranes (Yu et al., 2012). We have shown that LC3 can also become conjugated to phosphatidylserine (PS)-enriched membranes during non-canonical autophagy. ATG4D, but not the other paralogs, appears to be uniquely capable for the de-conjugation of LC3-PS *in vitro* (Durgan et al., 2021).

**Figure 4:**
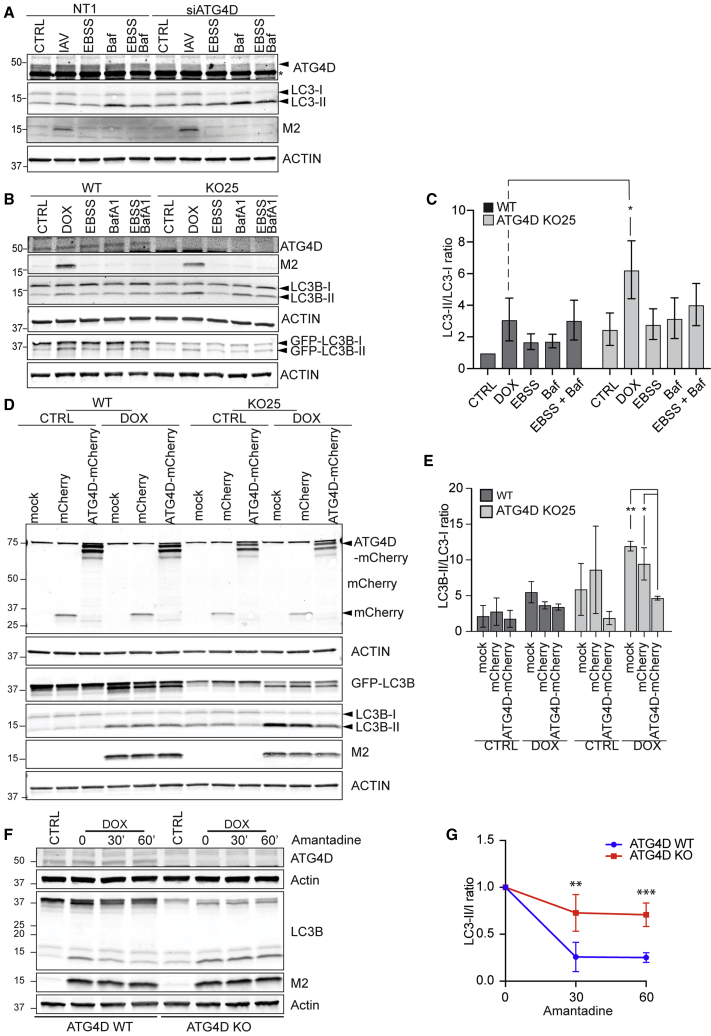
ATG4D depletion enhances LC3B lipidation (A) HCT116 cells were treated with siRNA as indicated and LC3B lipidation was analyzed after incubation for 8 h with PR8 (MOI of 20 PFU per cell), 2 h with EBSS, 1 h with 200 nM bafilomycin A1, or EBSS in combination with bafilomycin A1. Arrowhead indicates ATG4D-specific band. ^∗^Background band. (B) Tet-ON M2 WT and ATG4D KO clone 25 were treated with dox or as in (A). (C) Quantification of the experiment in (B). The graph shows the change in the LC3II/LC3I ratio from four independent experiments; bars show average ± SD. ^∗^p < 0.05. (D) WB analysis of Tet-ON M2 WT and ATG4D KO cells stably expressing mCherry-ATG4D or mCherry treated with dox. (E) Quantification of (D). The graph shows the change in the LC3II/LC3I ratio from three independent experiments; bars show mean ± SD. ^∗^p < 0.05, ^∗∗^p < 0.01. (F) M2 expression was induced in Tet-ON M2 WT and ATG4D^−/−^ clone 25 cells for 16 h followed by treatment with amantadine for 30 or 60 min. LC3 lipidation was analyzed by WB. (G) LC3II/LC3-I ratio of (F) was quantified and normalized to t = 0; average ± SD. ^∗∗^p < 0.01, ^∗∗∗^p < 0.001.

Depletion of ATG4D in HCT116 cells using small interfering RNA (siRNA) results in a slight increase in LC3 lipidation in fed conditions (Figure 4A). Levels of LC3B-II were also higher in IAV-infected and amino acid-starved cells (Earle’s balanced salt solution [EBSS]-treated) with reduced ATG4D levels compared to LC3B-II levels in control cells. We noticed a similar increase in LC3B-II levels in several clones of HCT116 ATG4D^−/−^ cells (Figure 4B; Figure S6A). Levels of M2-induced LC3B-II were significantly higher in ATG4D^−/−^ cells compared to WT (Figure 4C).

The different paralogs of ATG4 have been reported to exhibit some preference for individual ATG8 paralogs, and it has been suggested that the GABARAP subfamily is particularly susceptible to changes in delipidation conditions (Kauffman et al., 2018). We observed similar increases in levels of lipidation of GABARAP-L1 and GABARAP-L2 in ATG4D^−/−^ cells (Figure S6B).

In ATG4D^−/−^, basal and M2-induced LC3B lipidation levels were rescued upon expression of ATG4D-mCherry, but not in cells expressing the mCherry control plasmid (Figures 4D and 4E), further validating that ATG4D depletion results in increased levels of lipidated LC3. Moreover, delipidation of LC3-II under conditions where generation of new LC3-II is inhibited, i.e., in the presence of the M2 ion channel inhibitor amantadine, proceeded significantly more slowly in the absence of ATG4D (Figures 4F and 4G). ATG4D therefore plays a role in the delipidation of LC3/GABARAP-like molecules during non-canonical autophagy.

## Discussion

The subtractive CRISPR screen described herein correctly identified ATG7, ATG10, ATG3, and the ATG16L1-ATG5-ATG12 complex, all of which are known to be enzymatically required for LC3 lipidation in any context. In contrast, sgRNAs targeting genes such as FAM208A and MAX, both required for expression of the EGFP-LC3 transgene, provide strong signals on both individual screens but are not detected in the subtraction results (Figure S1, compare A and B). All sgRNAs targeting these genes lie close to the y = x line on a z-z plot (Figure 1E), demonstrating the specificity of the subtraction approach.

Although our screen was not optimized to detect genes essential for cell viability, we nonetheless identified a v-ATPase subunit. We previously identified the WD40 CTD of ATG16L1 as required for LC3 lipidation in response to M2 proton channel activity, ionophores, and during LAP (Fletcher et al., 2018). The recently described v-ATPase/ATG16L1 interaction identified during *Salmonella* infection also depends on this domain (Xu et al., 2019). This suggested that M2- and ionophore-induced LC3 lipidation might depend on the same mechanism. The antagonism of these processes by the *Salmonella* effector SopF and the concomitant association of ATG16L1 and v-ATPase suggest that this is the predominant mechanism by which non-canonical lipidation of LC3 and other ATG8-like molecules occurs. Interestingly, while this manuscript was in preparation, a link between cGAS-STING signaling-induced LC3 lipidation was shown to also depend on this ATG16L1/v-ATPase signaling axis (Fischer et al., 2020). This further highlights the importance of this mechanism in innate immune signaling. The v-ATPase consists of many subunits that assemble into the transmembrane V_O_ subcomplex and the cytosolic V_1_ subcomplex. Further experiments are required to define a precise role for the v-ATPase assembly state in ATG16L1 recruitment.

We identified the RalGAP complex as important for M2-induced LC3 lipidation. We were able to demonstrate that lack of either RalGAPβ alone or RalGAPα1 and RalGAPα2 expression inhibits LC3 lipidation caused by M2 expression. In the context of IAV infection the observed reduction in LC3 lipidation was accompanied by a modest reduction in M2 expression levels, although this did not substantially impair viral replication. No phenotype was observed in the other WD40 CTD-dependent LC3 lipidation pathways, indicating that an effect of RalGAP signaling could be M2 specific. The α subunit of the RalGAP complex contributes a conserved Asn side chain, the so-called “asparagine-thumb,” N1903 in RalGAPα1, to the active side of RalA or RalB, thereby increasing the rate of GTP hydrolysis and inactivating RalA/B (Chen et al., 2011; Shirakawa et al., 2009). Among other functions, RalA/B have been implicated in the inhibition of endocytosis and stimulation of exocytosis (Jullien-Flores et al., 2000; Moskalenko et al., 2002; Nakashima et al., 1999). Interestingly, we noted reduced intracellular M2 accumulation in RalGAP KO cells (Figure S2B), which was independent of LC3 lipidation.

The subtractive screen identified ATG4D as a negative regulator of LC3 lipidation, and ATG4D KO indeed resulted in increased levels of lipidated LC3B and GABARAPL1 and L2. The contributions of the individual ATG4 paralogs to priming and delipidation of ATG8s are still not fully understood. Paralogs appear to exhibit a preference for priming and/or delipidation of individual ATG8s. However, other paralogs appear to be able to compensate for loss of individual paralogs to some degree (Agrotis et al., 2019; Kauffman et al., 2018). ATG4D is the least well-characterized paralog, in part due to low *in vitro* activity of bacterially expressed protein (Kauffman et al., 2018). Elsewhere, we describe a role for ATG4D in delipidation of PS-conjugated LC3, which accumulates during WD40 CTD-dependent LC3 lipidation, including in response to IAV M2, but not during canonical autophagy (Durgan et al., 2021). While ATG4D exhibited a higher propensity to delipidate PS-conjugated ATG8s than ATG4B *in vitro* in that study, ATG4B could delipidate ATG8-PS in cells. In our system, ablation of ATG4D expression resulted in increased LC3B-II in the context of M2-induced LC3 lipidation, as well as under basal conditions and when canonical autophagy was induced. Similar observations were made for GABARAP-L1 and GABARAP-L2. Starvation-induced autophagy results in ATG8 conjugation exclusively to PE (Durgan et al., 2021; Ichimura et al., 2000). Thus, other factors, such as recruitment to specific compartments or regulation by post-translational modification of individual ATG4s, likely contribute to the increase in observed lipidated ATG8s. A missense mutation in ATG4D was found in Lagotto Romagnolo dogs with progressive neurological symptoms (Kyöstilä et al., 2015). These dogs exhibited neural lesions and intense vacuolization secretory tissues as well as increased levels of LC3-II in basal and starvation-induced autophagy (Kyöstilä et al., 2015; Syrjä et al., 2017). Whether the neurological phenotype is linked to reduced recycling of PS-conjugated ATG8 or a defect in ATG4D recruitment and/or trafficking remains to be determined. However, the observed phenotype highlights the importance of ATG4D *in vivo*.

Our data are consistent with a model in which recruitment of ATG16L1 by the v-ATPase targets lipidation of LC3 to erroneously neutral compartments. Targeting of this pathway by diverse pathogens such as *Salmonella* and influenza suggests it may represent an important damage detection mechanism, as even minimal damage to a compartment would compromise the ability of the v-ATPase to maintain proton gradients.

### Limitation statement

Although our subtractive CRISPR screen method correctly identified all of the known enzymatic components of the LC3 lipidation machinery, as well as some novel regulatory factors, it will not have exhaustively discovered all of the components of this pathway. Redundant elements will never be identified in this kind of screen, and genes essential for viability are also much harder to identify. The v-ATPase, required for cell viability, emerged as central to the regulation of non-canonical LC3 lipidation. Screens targeting the v-ATPase in influenza virus infections are also hampered by its requirement for virus entry. We chose to use an inducible M2 expression system that avoids this problem but consequently cannot account for a potential effect of other virus components on this pathway. M2-induced LC3 lipidation (unusually among non-canonical LC3 lipidation pathways that depend on ATG16L1 WD40 CTD) results in accumulation of LC3 at the plasma membrane in addition to intracellular vesicles. Our detergent-based FACS screen would not identify factors resulting in changes to the localization of lipidated LC3. Therefore, our screen will likely have missed genes that are either essential for cell viability, redundant, or change the targeting but not absolute amount of non-canonical LC3 lipidation.

## STAR★Methods

### Key resources table

**Table:** 

REAGENT or RESOURCE	SOURCE	IDENTIFIER
**Antibodies**		

anti-LC3B	Novus	Cat#NB100-2220, RRID:AB_10003146
anti-LC3B	Novus	Cat#NBP2-46892, RRID:AB_2891060
Mouse anti-Influenza A Virus M2 Protein antibody [14C2]	Abcam	Cat#ab5416, RRID:AB_304873
Rabbit anti-Influenza A Virus M2 Protein antibody	GeneTex	Cat#GTX125951, RRID:AB_11170983
Mouse anti-Influenza A Virus Nucleoprotein	Abcam	Cat#ab20343, RRID:AB_445525
Rabbit anti-Influenza A Virus Nucleoprotein	GeneTex	Cat#GTX125989, RRID:AB_11168364
anti-GABARP-L1	Proteintech	Cat#11010-1-AP, RRID:AB_2294415
anti-GABARP-L2	Abcam	Cat#ab126607, RRID:AB_11130165
anti-RalGAPα1	Abcam	Cat#ab182570, RRID:AB_2891061
anti-RalGAPβ	Abcam	Cat#ab151139, RRID:AB_2891062
anti-ATG4D	Proteintech	Cat#16924-1-AP, RRID:AB_2062024
anti-mCherry	Novus	Cat#NBP1-96752, RRID:AB_11034849
anti-ATG16L1	Cell Signaling Technology	Cat#8089, RRID:AB_10950320
anti-ATG16L1	MBL International	Cat#PM040, RRID:AB_1278757
anti-ATG12	Santa Cruz Biotechnology	Cat#sc271688, RRID:AB_10709301
anti-ATP6V1D	Abcam	Cat#ab157458, RRID:AB_2732041
anti-ATP6V1A	Abcam	Cat#ab199326, RRID:AB_2802119
anti-ATP6V1B1/2	Santa Cruz Biotechnology	Cat#sc55544, RRID:AB_831844
anti-FLAG	Sigma-Aldrich	Cat#F1804, RRID:AB_262044
anti-β-Actin	Proteintech	Cat#20536-1-AP, RRID:AB_10700003
anti-β-Actin	Proteintech	Cat#66009-1, RRID:AB_2687938
anti-GAPDH	Abcam	Cat#ab8245, RRID:AB_2107448
anti-tubulin	Bio-Rad	Cat#MCA77G, RRID:AB_325003
donkey-anti-mouse-IgG-Alexa568	Thermo	Cat#A10037, RRID:AB_2534013
goat anti-mouse-IgG-Alexa647	Thermo	Cat#A32728, RRID:AB_2633277
IRDye 680RD Goat anti-Mouse IgG	LI-COR Biosciences	Cat#926-68070, RRID:AB_10956588
IRDye 680RD Goat anti-Rabbit IgG	LI-COR Biosciences	Cat#926-68071, RRID:AB_10956166
IRDye 800CW Goat anti-Mouse IgG	LI-COR Biosciences	Cat#926-32210, RRID:AB_621842
IRDye 800CW Goat anti-Rabbit IgG	LI-COR Biosciences	Cat#926-32211, RRID:AB_621843
IRDye 680RD Goat anti-Rat IgG	LI-COR Biosciences	Cat#926-68076, RRID:AB_10956590

**Chemicals, peptides, and recombinant proteins**		

Torin 1	Selleckchem	Cat#S2827
VPS34 inhibitor 1	Selleckchem	Cat#S8456
Doxycycline	SIGMA	Cat#D9891
Monensin	SIGMA	Cat#M5273
Wortmannin	SIGMA	Cat#W1628
amantadine hydrochloride	SIGMA	Cat#A1260
Bafilomycin A1	Abcam	Cat#ab120497
Polybrene	Santa Cruz	Cat#sc134220
G418	Invivogen	Cat#ant-gn
Blasticidin	Invivogen	Cat#ant-bl
Puromycin	Invivogen	Cat#ant-pr
TPCK-trypsin	Worthington	Cat#LS003750
HiFi Cas9 Nuclease V3	IDT	Cat#1081060

**Critical commercial assays**		

Zombie Violet Fixable Viability Kit	Biolegend	Cat#423114
QIAamp DNA FFPE Tissue Kit	QIAGEN	Cat#56404
RNeasy Mini Kit	QIAGEN	Cat#74134
SuperSCRIPT-II reverse transcriptase	ThermoFisher Scientific	Cat#18064014
Dynabeads Protein A	Invitrogen	Cat#10002D
Dynabeads Protein G	Invitrogen	Cat#10004D
Lipofectamine RNAiMAX	ThermoFisher Scientific	Cat#13778075
LR clonase	ThermoFisher Scientific	Cat#11791020

**Experimental models: Cell lines**		

HEK293T	ECACC	Cat#12022001; RRID:CVCL_0063
HCT-116	ATCC	Cat#CCL-247; RRID:CVCL_0291
HCT-116 ATG16L1−/− #E9	Fletcher et al., 2018	N/A
HCT-116 EGFP-LC3B TetON-M2	this study	N/A
HCT-116 EGFP-LC3B TetON-M2 RalGAPB −/− #A4	this study	N/A
HCT-116 EGFP-LC3B TetON-M2 RalGAPB −/− #B8	this study	N/A
HCT-116 EGFP-LC3B TetON-M2 RalGAPA1/A2 KO #1	this study	N/A
HCT-116 EGFP-LC3B TetON-M2 ATG4D KO #25	this study	N/A
HCT-116 EGFP-LC3B TetON-M2 ATG4D KO #25 mCherry	this study	N/A
HCT-116 EGFP-LC3B TetON-M2 ATG4D KO #25 ATG4D-mCherry	this study	N/A
HCT-116 EGFP-LC3B TetON-M2 mCherry	this study	N/A
HCT-116 EGFP-LC3B TetON-M2 mCherry-SopF	this study	N/A
HeLa	ECACC	Cat#93021013; RRID:CVCL_0030
A549	NCI-DTP	Cat#A549; RRID:CVCL_0023

**Oligonucleotides**		

random hexamer primer	Roche	Cat#11034731001
qPCR primers for RalGAPα2, GCCTGGATAACCAGTCTTCTCC and CACAGATCAGCCTGTAGGCTTG	This study	Cat#N/A
qPCR primers for actin, GGGGTGTTGAAGGTCTCAAA and TTCTACAATGAGCTGCGTGTG	This study	Cat#N/A
CRISPR guide RALGAPB_A, GTAAGCATAGTCGAATCTGAC	Shalem et al., 2014	N/A
RALGAPB_B, GCTATGGACTGACCCTTCCAT	Shalem et al., 2014	N/A
CRISPR guide RALGAPA1, GACTTCTTCACGTCCCCGTG	This study	Cat#N/A
CRISPR guide RALGAPA2, GTGGACTTCTTCACATCCCCG	This study	Cat#N/A
CRISPR guide ATG4D guide 1, ggcgggacacaaagucccgc	Synthego	Cat#N/A
CRISPR guide ATG4D guide 2, GGGACUUUGUGUCCCGCCUG	Synthego	Cat#N/A
CRISPR guide ATG4D guide 3, Cccggcgguaugugagccac	Synthego	Cat#N/A
CRISPR guide ATG16L1 guide 1, CAAUUUAGUCCCGGACAUGA	Synthego	Cat#N/A
CRISPR guide ATG16L1 guide 2, GUCCCGGACAUGAUGGCACA	Synthego	Cat#N/A
siGENOME Human ATG4D siRNA smart pool	Horizon	Cat#M-005790-01-0005
siGENOME Non-Targeting Control siRNA Pool #1	Horizon	Cat#D-001206-13-20

**Recombinant DNA**		

pInd10b-Ud-M2	this study	N/A
M5PmCherry-hATG16L1	R.U., unpublished data	N/A
M5P-mCherry-SopF	this study	N/A
M5P-mCherry control	this study	N/A
pBABE-FLAG-S-mATG16L1	Gammoh et al., 2013	N/A
pBABE-FLAG-S-mATG16L1[K490A]	Fletcher et al., 2018	N/A
plenti-ATG4D-mCherry-hygR	this study	N/A
pENTR-ATG4D	Transomics	Cat#HQ447598;
pLenti-GWT-mCherry-HygR	F. Sorgeloos, personal communication	N/A
pLenti-PGK-RalGAPA1-hygB	This study	N/A
pLenti-CMV- RalGAPA1-hygB	This study	N/A
pLenti-mCherry-RalGAPA1	This study	N/A
pENTR-RalGAPA1	Transomics	Cat#BC168361;
pLenti-PGK-GWT-hygB	F. Sorgeloos, personal communication	N/A
pLenti CMV hygro DEST	Addgene	Cat#17454; Addgene_17454
pLenti-mCherry-GWT	F. Sorgeloos, personal communication	N/A
Human GeCKOv2 CRISPR knockout pooled library	Addgene	Cat#1000000049;
lenti-Cas9-Blast	Addgene	Cat#52962; Addgene_52962

**Software and algorithms**		

Fiji	Rueden et al., 2017; Schindelin et al., 2012	RRID:SCR_002285
Prism 9	GraphPad Software, LLC	RRID:SCR_002798
Image Studio Lite	LI-COR Biosciences	RRID:SCR_013715

### Resource availability

#### Lead contact

Further information and requests for resources and reagents should be directed to and will be fulfilled by the Lead Contact, Rupert Beale (Rupert.beale@crick.ac.uk).

#### Materials availability

Plasmids and cell lines generated in this study are available from the lead contact with a completed Materials Transfer Agreement.

### Experimental model and subject details

HCT116 cells are a male human colon carcinoma cell line with epithelial-like morphology. HEK293t cells are a human embryonic kidney cell line with epithelial-like morphology that express the SV40 large T antigen. This study uses influenza A virus strains PR8 (strain A/Puerto Rico/8/1934) and MUd, a reassortant PR8 variant carrying segment 7 of IAV strain A/Udorn/307/1972 (Noton et al., 2009).

### Method details

#### Cell culture and RNA interference

HCT116 cells were cultured in McCoys 5A medium (Lonza) supplemented with 5% fetal calf serum (FCS). HEK293t and MDCK-II (a kind gift from P. Digard, Roslin Institute, Edinburgh, UK) were cultured in Dulbecco’s modified Eagle’s medium (DMEM; GIBCO Life Technologies) containing 10% FCS. HCT116 EGFP-LC3B TetON M2 (Tet ON M2) express the IAV strain Udorn M2 protein under a doxycycline (dox) inducible promoter. It was generated by transducing HCT116 EGFP-LC3B cells using a lentivirus produced with pInd10b-Ud-M2 followed by initial selection with G418. A high expressing cell clone was isolated using FACS. Unless indicated otherwise 10 μg/ml dox for 8 or 16 h was used to induce expression of M2. All cells were maintained in an incubator at 37°C with 5% CO2. For siRNA experiments, cells were treated with 40 nM of non-targeting (NT1) or target-specific siRNA oligonucleotides (Dharmacon On-Target Plus Smart Pool), using Lipofectamine RNAi-MAX (Invitrogen) according to manufacturer’s instructions.

#### Plasmids

M4P-EGFP-LC3B and pOPG was a kind gift from F. Randow. pMD2.G (http://addgene.org/12259) and psPAX2 (http://addgene.org/12260) were a gift from Didier Trono. pSpCas9(BB)-2A-Puro (PX459) V2.0 was a gift from Feng Zhang (http://addgene.org/62988) (Ran et al., 2013).

The insert encoding the M2 protein from IAV virus strain Udorn was generated by overlap extension PCR using pHW2000-seg7-Ud as a template and cloned into pInd10b-HA-KRAS using AgeI and MluI sites by standard restriction ligation cloning.

M5P-mCherry-SopF was subcloned into M5PmCherry-hATG16L1 (R. Ulferts, unpublished data) from M4P-EGFP-SopF (a kind gift from F. Randow) using the PciI and NotI restriction sites.

M5P-mCherry control vector was generated by inserting a stop codon downstream of mCherry by inserting a linker generated by annealing 5′-CATGTCGTAAGTAATTAAGC-3′ and 5′-GGCCGCTTAATTACTTACGA-3′ into the PciI and NotI restriction sites of M5P-mCherry-hATG16L1.

pLenti-ATG4D-mCherry-hygR was generated by gateway cloning with LR clonase using pENTR-ATG4D and pLenti-GWT-mCherry-HygR.

pLenti-PGK-RalGAPA1-hygB, pLenti-CMV-RalGAPA1-hygB, and pLenti-mCherry-RalGAPA1 were generated by gateway cloning with LR clonase using pENTR-RalGAPA1 and pLenti-PGK-GWT-hygB, pLenti-CMV-hygro-DEST or pLenti-mCherry-GWT.

#### Whole-genome CRISPR screen

Human GeCKOv2 CRISPR knockout pooled library and lenti-Cas9-Blast was a gift from Feng Zhang Sanjana et al., 2014. The library was amplified and the lentivirus library generated as described in Shalem et al., 2014. HCT-116 stably expressing EGFP-LC3B tet-ON M2 were transduced with cas9-blast lentivirus and selected with blasticidin. Cells were transduced with the virus library at a 300x library representation at an moi of 0.3 viruses per cell, followed by selection with puromycin. Selected cells were passaged at 300x library representation for 14 days. Expression of M2 was induced by addition of doxycycline. 16 h post induction cells were harvested and stained for M2 surface expression using anti-M2 antibody (14C2) followed by staining with donkey-anti-mouse-IgG-Alexa568 (Thermo). Cells were then permeabilised with 0.1% saponin (Sigma) in PBS or mock treated. Zombie-violet (Biolegend, 423114, 1:200) staining was used to exclude dead cells. Cells were fixed with 1% formaldehyde in PBS prior to sorting. The top and bottom 10% EGFP-expressing cells of the M2-positive cell populations were collected on a BD FACSAria™ Fusion or BD FACSJazz™ instrument. Genomic DNA was isolated using the QIAamp DNA FFPE Tissue Kit (QIAGEN) essentially as described in the manufacturers protocol except that buffer ATL was supplemented with 300 mM NaCl and the cell lysate incubated for 2 h at 56°C under constant agitation. PCR amplification was carried out as described in Shalem et al., 2014. Illumina Hiseq was performed at the Bauer sequencing facility, Harvard.

#### Data analysis of the CRISPR screen

For analysis of the screen data, we devised a simple but robust subtraction approach based on our previous method for genome-wide screen analysis (Li et al., 2020). Briefly, read counts corresponding to each guide RNA were normalized to reads per million and and log transformed. Quantile normalization was performed in R version 3.6.1. In comparisons between intervention and control experiments, over/under-representation was quantified as the distance from the expected null (i.e., the y=x line on a plot of read counts.) In order to control for heteroscedasticity, these distances were normalized to local z-scores calculated for sliding bins of adjacent read count results (Li et al., 2020).

In order to remove the background effects of specific genes required for expression of the EGFP construct, z-scores from the background (EGFP expression) screen were then subtracted from z-scores for the saponin-permeabilisation (M2) screen. p-values were calculated from the sum of z-scores for sgRNAs targeting a particular gene compared to a density function modeled on an empirical distribution of possible combinations of sgRNA z-scores permuted at least 1e8 times by randomly rearranging z-scores for all sgRNAs in the screen.

#### CRISPR knockout of single genes

Stable knock out cell lines using CRISPR technology were generated using either plasmid or nucleofection with guide RNAs (Synthego) and Cas9 (Thermo). Single cell clones were selected and absence of gene expression confirmed by western blotting or qPCR.

**Table:** 

Name	guide sequence
RALGAPB_A	GTAAGCATAGTCGAATCTGAC
RALGAPB_B	GCTATGGACTGACCCTTCCAT
RALGAPA1	GACTTCTTCACGTCCCCGTG
RALGAPA2	GTGGACTTCTTCACATCCCCG
ATG4D guide 1	ggcgggacacaaagucccgc
ATG4D guide 2	GGGACUUUGUGUCCCGCCUG
ATG4D guide 3	Cccggcgguaugugagccac
ATG16L1	CAAUUUAGUCCCGGACAUGA
	GUCCCGGACAUGAUGGCACA
	Both guides were transfected together.

#### qPCR

RNA was isolated using RNAeasy extraction Kit (QIAGEN) following manufacturer instructions. cDNA was synthesized using SuperSCRIPT-II reverse transcriptase and random hexamer primer according to manufactures protocol. qPCR was performed using taq PCR and cycler using primers GCCTGGATAACCAGTCTTCTCC and CACAGATCAGCCTGTAGGCTTG for RalGAPα2 and GGGGTGTTGAAGGTCTCAAA and TTCTACAATGAGCTGCGTGTG for actin.

#### Influenza A virus production

Stocks of influenza A virus PR8 (strain A/Puerto Rico/8/1934) and MUd, a reassortant PR8 variant carrying segment 7 of IAV strain A/Udorn/307/1972 (Noton et al., 2009) were generated using the eight plasmid-based systems as previously described (de Wit et al., 2004) and propagated on MDCK-II cells in presence of TPCK-trypsin (Worthington). For infection, cells were first washed with serum free medium and incubated with virus in serum-free medium at 37°C. After 1 h, the medium was replaced with DMEM containing 1% FCS. Virus titers were determined by plaque assay on MDCK-II cells.

#### Retrovirus and lentivirus production

Retrovirus and lentivirus particles were generated using packaging plasmids MD2-G and pOPG or psPAX2, respectively, by transfecting HEK293T using PEI. Cells were transduced with virus by spinfection in the presence of 8 μg/ml of polybrene followed by selection with G418, blasticidin, puromycin or fluorescence-assisted cell sorting as appropriate.

#### Entosis assay

The entosis assay was carried out as described previously (Florey et al., 2011). HCT116 wild-type or knock out cells stably expressing EGFP-LC3B were grown in 35 mm glass bottom dishes and imaged every 4 min for 20 h. DIC and fluorescent images were acquired using a confocal Zeiss LSM 780 microscope (Carl Zeiss Ltd) equipped with a 40x oil immersion 1.40 numerical aperture (NA) objective using Zen software (Carl Zeiss Ltd).

#### Western blotting

Cells were lyses in ice cold RIPA (10 mM Tris–HCl pH 7.5, 150 mM NaCl, 1% Triton X-100, 0.1% SDS, 1% sodium deoxycholate) or NP40 buffer (0.5% NP-40, 25 mM Tris–HCl pH 7.5, 100 mM NaCl, 50 mM NaF) supplemented with Complete, Mini, EDTA-free Protease Inhibitor Cocktail (11836170001). Lysates were cleared by centrifugation and the protein concentration determined using BCA assay (Pierce) and IgG or BSA as a standard. Proteins were separated in a Mini-PROTEAN®TGX gel (Bio-Rad) and transferred onto nitrocellulose. After blocking with 5 or 10% dry milk powder in TBS supplemented with 0.1% Tween 20, blots were incubated for 1 h to overnight with primary antibody at the indicated dilution, followed by the appropriate species specific IRdye 800CW and 680LT coupled secondary antibodies (LICOR) and imaged using an Odyssey CLx scanner (Li-COR). In some cases, antibodies were subsequently removed using Restore Stripping buffer (ThermoFisher Scientific; #21059) according to manufacturer’s instructions, reblocked and incubated with primary and secondary antibody.

#### Immunofluorescence

Cells were grown on coverslips pre-treated with 0.001% poly-L-lysine (Sigma), fixed using 4% formaldehyde in PBS, permeabilised with 0.05% saponin and incubated with primary antibody, prior to staining with AlexaFluor 405, 488, 568 or 647 coupled secondary antibodies. Images were acquired using Zeiss LSM800 with Airyscan and further processed using Adobe Photoshop CC2020 or Fiji 1.0 (Rueden et al., 2017; Schindelin et al., 2012).

#### Immunoprecipitation (IP)

This protocol was adapted from Xu et al. (2019). Two 15cm dishes of cells were washed twice with ice-cold PBS and lysed in a buffer containing 50 mM Tris-HCl (pH 7.5), 150 mM NaCl, 2 mM EDTA, 0.8% C_12_E_9_ (Sigma-Aldrich, P9641) and protease inhibitors (Roche, 11836170001 or Sigma-Aldrich, P8340). Lysates were centrifugated at 13,000 rpm, 4°C for 30 minutes and the pellet discarded. A small amount of lysate was removed for western blotting. 60 μL of Dynabeads Protein A or Protein G (Invitrogen, 10002D and 10004D) were incubated with 5 μL of IP antibody for 30 minutes rotating at 4°C. Beads were washed once in lysis buffer before incubation with lysate for 2h rotating at 4°C. Beads were then washed five times in a buffer containing 50 mM Tris-HCl (pH 7.5), 150 mM NaCl, 2 mM EDTA, 1% Triton X-100, and 0.1% C_12_E_9_. Bound proteins were eluted by boiling at 95°C for 5 minutes in SDS-loading buffer. Eluted samples were probed by western blot as described above.

### Quantification and statistical analysis

Analysis and quantification of western blots were carried out using Imagestudio light (Li-COR). Unless indicated otherwise in the respective figure legend, the mean ± SD of three independent experiments is shown and the significance was analyzed using unpaired Student’s t test (GraphPad Prism 9 software, San Diego, CA, USA).

## Data Availability

• All the raw data reported in this paper will be shared by the lead contact upon request. • This paper does not report original code. • Any additional information required to reanalyze the data reported in this paper is available from the lead contact upon request.

## Supplemental information

Document S1. Figures S1–S6

Document S2. Article plus supplemental information

## References

[bib1] Agrotis A., Pengo N., Burden J.J., Ketteler R. (2019). Redundancy of human ATG4 protease isoforms in autophagy and LC3/GABARAP processing revealed in cells. Autophagy.

[bib2] Beale R., Wise H., Stuart A., Ravenhill B.J., Digard P., Randow F. (2014). A LC3-interacting motif in the influenza A virus M2 protein is required to subvert autophagy and maintain virion stability. Cell Host Microbe.

[bib3] Chen X.-W., Leto D., Xiong T., Yu G., Cheng A., Decker S., Saltiel A.R. (2011). A Ral GAP complex links PI 3-kinase/Akt signaling to RalA activation in insulin action. Mol. Biol. Cell.

[bib4] Ciampor F., Bayley P.M., Nermut M.V., Hirst E.M.A., Sugrue R.J., Hay A.J. (1992). Evidence that the amantadine-induced, M2-mediated conversion of influenza A virus hemagglutinin to the low pH conformation occurs in an acidic trans Golgi compartment. Virology.

[bib41] de Wit E., Spronken M.I., Bestebroer T.M., Rimmelzwaan G.F., Osterhaus A.D., Fouchier R.A. (2004). Efficient generation and growth of influenza virus A/PR/8/34 from eight cDNA fragments. Virus Res.

[bib5] Durgan J., Lystad A.H., Sloan K., Carlsson S.R., Wilson M.I., Marcassa E., Ulferts R., Webster J., Lopez-Clavijo A.F., Wakelam M.J. (2021). Non-canonical autophagy drives alternative ATG8 conjugation to phosphatidylserine. Mol. Cell.

[bib6] Eng K.E., Panas M.D., Karlsson Hedestam G.B., McInerney G.M. (2010). A novel quantitative flow cytometry-based assay for autophagy. Autophagy.

[bib7] Fischer T.D., Wang C., Padman B.S., Lazarou M., Youle R.J. (2020). STING induces LC3B lipidation onto single-membrane vesicles via the V-ATPase and ATG16L1-WD40 domain. J. Cell Biol..

[bib8] Fletcher K., Ulferts R., Jacquin E., Veith T., Gammoh N., Arasteh J.M., Mayer U., Carding S.R., Wileman T., Beale R., Florey O. (2018). The WD40 domain of ATG16L1 is required for its non-canonical role in lipidation of LC3 at single membranes. EMBO J..

[bib9] Florey O., Kim S.E., Sandoval C.P., Haynes C.M., Overholtzer M. (2011). Autophagy machinery mediates macroendocytic processing and entotic cell death by targeting single membranes. Nat. Cell Biol..

[bib10] Florey O., Gammoh N., Kim S.E., Jiang X., Overholtzer M. (2015). V-ATPase and osmotic imbalances activate endolysosomal LC3 lipidation. Autophagy.

[bib11] Fujioka Y., Suzuki S.W., Yamamoto H., Kondo-Kakuta C., Kimura Y., Hirano H., Akada R., Inagaki F., Ohsumi Y., Noda N.N. (2014). Structural basis of starvation-induced assembly of the autophagy initiation complex. Nat. Struct. Mol. Biol..

[bib39] Gammoh N., Florey O., Overholtzer M., Jiang X. (2013). Interaction between FIP200 and ATG16L1 distinguishes ULK1 complex-dependent and -independent autophagy. Nat Struct Mol Biol.

[bib12] Gannagé M., Dormann D., Albrecht R., Dengjel J., Torossi T., Rämer P.C., Lee M., Strowig T., Arrey F., Conenello G. (2009). Matrix protein 2 of influenza A virus blocks autophagosome fusion with lysosomes. Cell Host Microbe.

[bib13] Henkel J.R., Popovich J.L., Gibson G.A., Watkins S.C., Weisz O.A. (1999). Selective perturbation of early endosome and/or *trans*-Golgi network pH but not lysosome pH by dose-dependent expression of influenza M2 protein. J. Biol. Chem..

[bib14] Ichimura Y., Kirisako T., Takao T., Satomi Y., Shimonishi Y., Ishihara N., Mizushima N., Tanida I., Kominami E., Ohsumi M. (2000). A ubiquitin-like system mediates protein lipidation. Nature.

[bib15] Jacquin E., Leclerc-Mercier S., Judon C., Blanchard E., Fraitag S., Florey O. (2017). Pharmacological modulators of autophagy activate a parallel noncanonical pathway driving unconventional LC3 lipidation. Autophagy.

[bib16] Jullien-Flores V., Mahé Y., Mirey G., Leprince C., Meunier-Bisceuil B., Sorkin A., Camonis J.H. (2000). RLIP76, an effector of the GTPase Ral, interacts with the AP2 complex: Involvement of the Ral pathway in receptor endocytosis. J. Cell Sci..

[bib17] Kauffman K.J., Yu S., Jin J., Mugo B., Nguyen N., O’Brien A., Nag S., Lystad A.H., Melia T.J. (2018). Delipidation of mammalian Atg8-family proteins by each of the four ATG4 proteases. Autophagy.

[bib18] Kaufmann A., Beier V., Franquelim H.G., Wollert T. (2014). Molecular mechanism of autophagic membrane-scaffold assembly and disassembly. Cell.

[bib19] Kyöstilä K., Syrjä P., Jagannathan V., Chandrasekar G., Jokinen T.S., Seppälä E.H., Becker D., Drögemüller M., Dietschi E., Drögemüller C. (2015). A missense change in the *ATG4D* gene links aberrant autophagy to a neurodegenerative vacuolar storage disease. PLoS Genet..

[bib20] Li B., Clohisey S.M., Chia B.S., Wang B., Cui A., Eisenhaure T., Schweitzer L.D., Hoover P., Parkinson N.J., Nachshon A. (2020). Genome-wide CRISPR screen identifies host dependency factors for influenza A virus infection. Nat. Commun..

[bib21] Martin T.D., Chen X.-W., Kaplan R.E.W., Saltiel A.R., Walker C.L., Reiner D.J., Der C.J. (2014). Ral and Rheb GTPase activating proteins integrate mTOR and GTPase signaling in aging, autophagy, and tumor cell invasion. Mol. Cell.

[bib22] Mizushima N., Komatsu M. (2011). Autophagy: Renovation of cells and tissues. Cell.

[bib23] Mizushima N., Noda T., Yoshimori T., Tanaka Y., Ishii T., George M.D., Klionsky D.J., Ohsumi M., Ohsumi Y. (1998). A protein conjugation system essential for autophagy. Nature.

[bib24] Moskalenko S., Henry D.O., Rosse C., Mirey G., Camonis J.H., White M.A. (2002). The exocyst is a Ral effector complex. Nat. Cell Biol..

[bib25] Nakashima S., Morinaka K., Koyama S., Ikeda M., Kishida M., Okawa K., Iwamatsu A., Kishida S., Kikuchi A. (1999). Small G protein Ral and its downstream molecules regulate endocytosis of EGF and insulin receptors. EMBO J..

[bib40] Noton S.L., Simpson-Holley M., Medcalf E., Wise H.M., Hutchinson E.C., McCauley J.W., Digard P. (2009). Studies of an influenza A virus temperature-sensitive mutant identify a late role for NP in the formation of infectious virions. J Virol.

[bib26] Ran F.A., Hsu P.D., Wright J., Agarwala V., Scott D.A., Zhang F. (2013). Genome engineering using the CRISPR-Cas9 system. Nat. Protoc..

[bib27] Ren Y., Li C., Feng L., Pan W., Li L., Wang Q., Li J., Li N., Han L., Zheng X. (2015). Proton channel activity of influenza a virus matrix protein 2 contributes to autophagy arrest. J. Virol..

[bib28] Rueden C.T., Schindelin J., Hiner M.C., DeZonia B.E., Walter A.E., Arena E.T., Eliceiri K.W. (2017). ImageJ2: ImageJ for the next generation of scientific image data. BMC Bioinformatics.

[bib29] Sanjana N.E., Shalem O., Zhang F. (2014). Improved vectors and genome-wide libraries for CRISPR screening. Nat. Methods.

[bib30] Sanjuan M.A., Dillon C.P., Tait S.W.G., Moshiach S., Dorsey F., Connell S., Komatsu M., Tanaka K., Cleveland J.L., Withoff S., Green D.R. (2007). Toll-like receptor signalling in macrophages links the autophagy pathway to phagocytosis. Nature.

[bib31] Schindelin J., Arganda-Carreras I., Frise E., Kaynig V., Longair M., Pietzsch T., Preibisch S., Rueden C., Saalfeld S., Schmid B. (2012). Fiji: An open-source platform for biological-image analysis. Nat. Methods.

[bib32] Shalem O., Sanjana N.E., Hartenian E., Shi X., Scott D.A., Mikkelson T., Heckl D., Ebert B.L., Root D.E., Doench J.G., Zhang F. (2014). Genome-scale CRISPR-Cas9 knockout screening in human cells. Science.

[bib33] Shirakawa R., Fukai S., Kawato M., Higashi T., Kondo H., Ikeda T., Nakayama E., Okawa K., Nureki O., Kimura T. (2009). Tuberous sclerosis tumor suppressor complex-like complexes act as GTPase-activating proteins for Ral GTPases. J. Biol. Chem..

[bib34] Syrjä P., Anwar T., Jokinen T., Kyöstilä K., Jäderlund K.H., Cozzi F., Rohdin C., Hahn K., Wohlsein P., Baumgärtner W. (2017). Basal autophagy is altered in Lagotto Romagnolo dogs with an *ATG4D* mutation. Vet. Pathol..

[bib35] Tchasovnikarova I.A., Timms R.T., Matheson N.J., Wals K., Antrobus R., Göttgens B., Dougan G., Dawson M.A., Lehner P.J. (2015). Epigenetic silencing by the HUSH complex mediates position-effect variegation in human cells. Science.

[bib36] Xu Y., Zhou P., Cheng S., Lu Q., Nowak K., Hopp A.-K., Li L., Shi X., Zhou Z., Gao W. (2019). A bacterial effector reveals the V-ATPase-ATG16L1 axis that initiates xenophagy. Cell.

[bib38] Yu Z.Q., Ni T., Hong B., Wang H.Y., Jiang F.J., Zou S., Chen Y., Zheng X.L., Klionsky D.J., Liang Y., Xie Z. (2012). Dual roles of Atg8-PE deconjugation by Atg4 in autophagy. Autophagy.

[bib37] Zhirnov O.P., Klenk H.D. (2013). Influenza A virus proteins NS1 and hemagglutinin along with M2 are involved in stimulation of autophagy in infected cells. J. Virol..

