# Supplementary material for: Subtractive CRISPR screen identifies the ATG16L1/vacuolar ATPase axis as required for non-canonical LC3 lipidation
Source: Cell Rep. 2021 Oct 26;37(4):109899. doi: 10.1016/j.celrep.2021.109899 (PMC8567314; doi:10.1016/j.celrep.2021.109899)
Supplement: Document S1. Figures S1–S6 [file mmc1.pdf]

Cell Reports, Volume *37*

Supplemental information

Subtractive CRISPR screen identifies the ATG16L1/vacuolar ATPase axis as required for non-canonical LC3 lipidation

Rachel Ulferts, Elena Marcassa, Lewis Timimi, Liam Changwoo Lee, Andrew Daley, Beatriz Montaner, Suzanne Dawn Turner, Oliver Florey, John Kenneth Baillie, and Rupert Beale

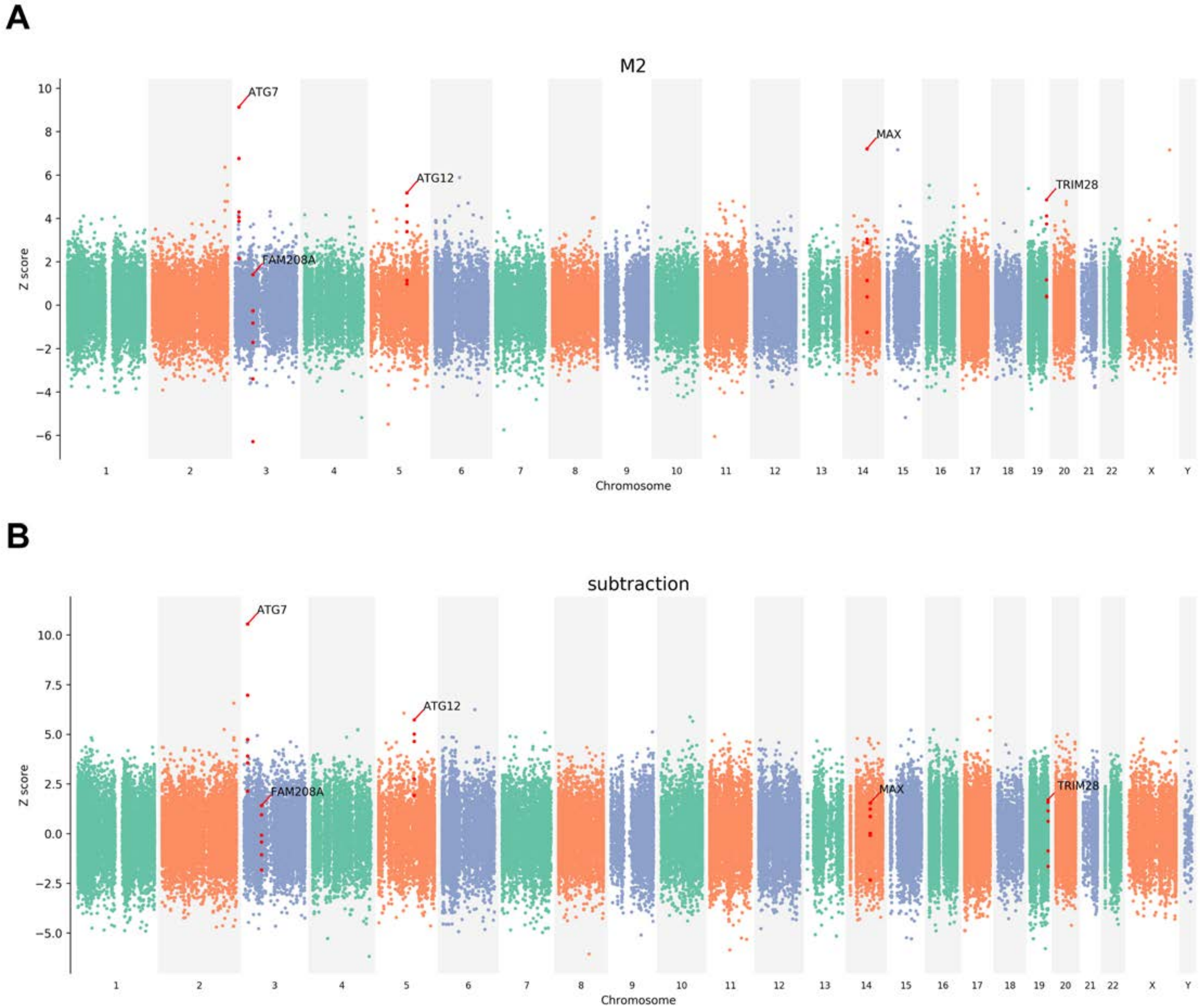

Figure S1, related to figure 1: Spatial distribution on the genome of the z-score of sgRNAs the A) permeabilised FACS sort of EGFP-LC3B levels and B) the subtractive analysis of permeabilised and unpermeabilised FACS sorts.

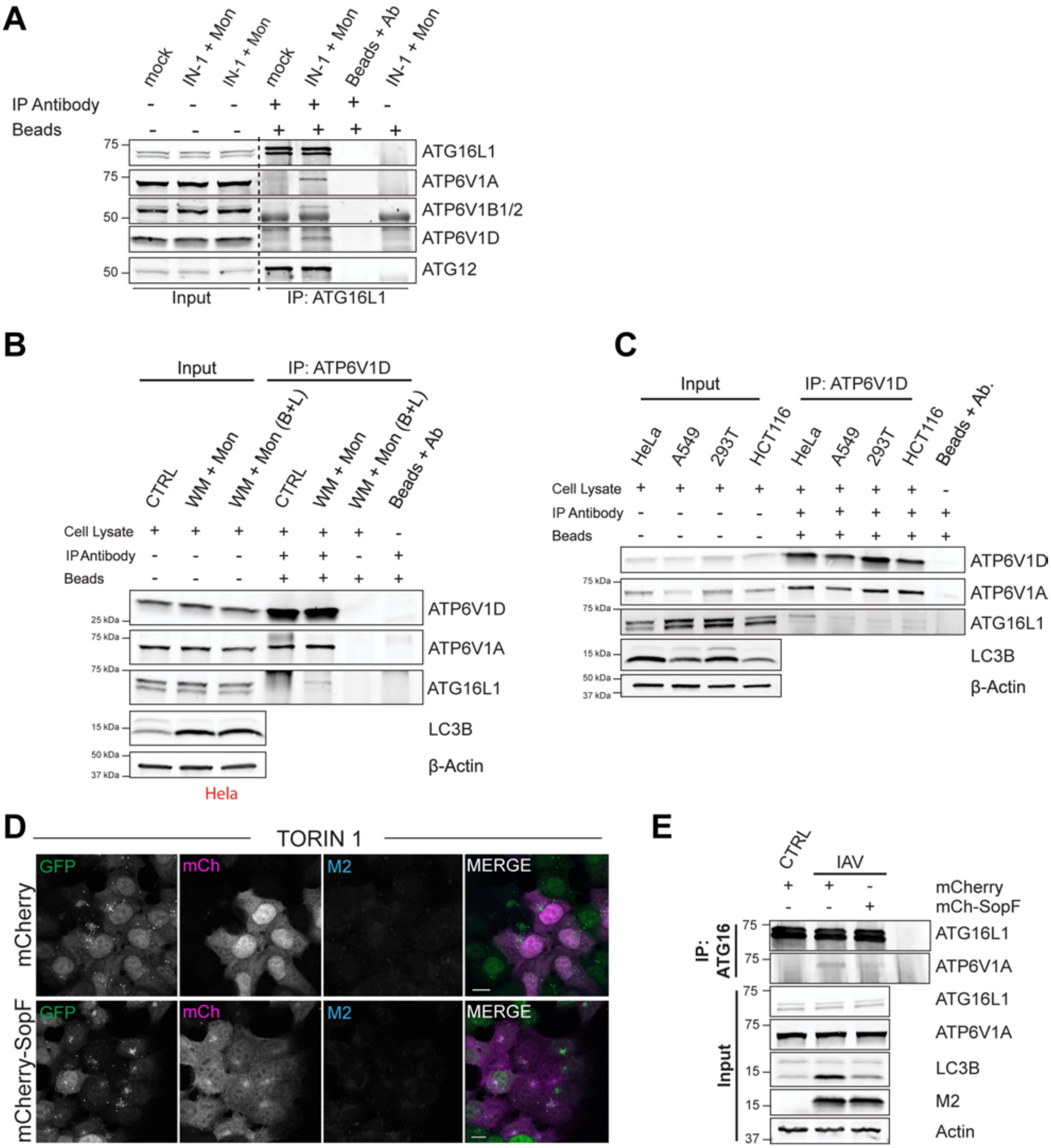

Figure S2, related to figure 2: A) Immunoprecipitation analysis of endogenous ATG16L1 in HeLa cells treated with VPS34 IN-1 (pretreatment: 1 µM for 30 minutes) followed by monensin (100 µM for 1 h). B) Immunoprecipitation analysis of endogenous of ATP6V1D in HeLa cells treated with wortmannin (IN-1 substitute; pretreatment: 100 nM for 30 minutes) followed by monensin (100 µM for 1h). C) Immunoprecipitation analysis of endogenous of ATP6V1D in the indicated cell lines following treatment with VPS34 IN-1 (pretreatment: 1 µM for 30 minutes) followed by monensin (100 µM for 1h). D) IF analysis of EGFP-LC3B relocalisation in HCT116 EGFP-LC3B TetON-M2 cells stably expressing mCherry or mCherry-SopF after treatment with Torin 1 (250 nM for 3 h). scale bar 10 µm F) Pull down analysis of ATG16L1 interaction with the vATPase in HCT116 EGFP-LC3B cells stably expressing mCherry or mCherry-SopF after infection with PR8 for 16 h.

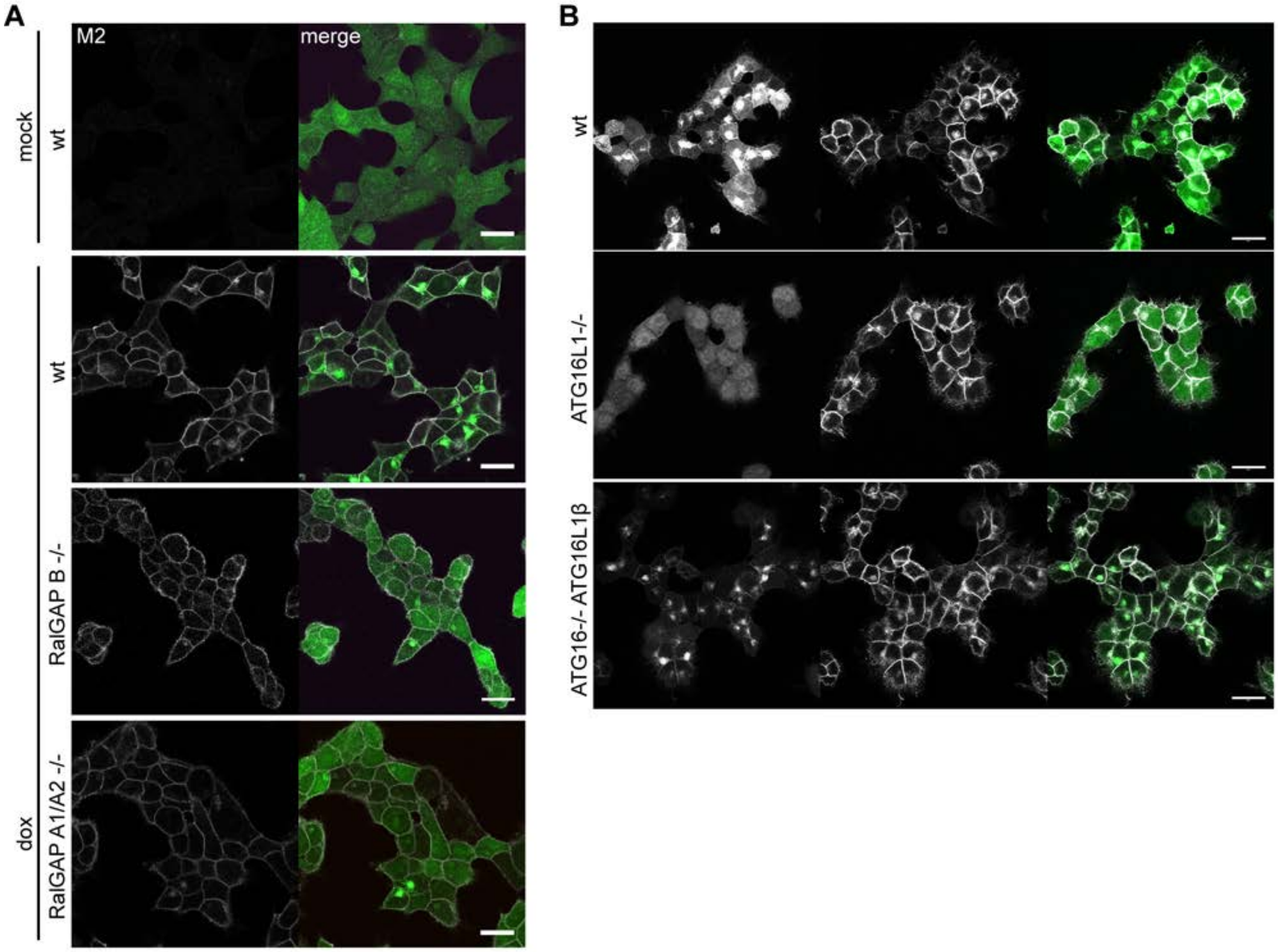

Figure S3, related to figure 3: A) IF analysis of M2 localisation in HCT116 EGFP-LC3B TetON-M2 wt or RalGAPA1A2-/- or RalGAPB -/- cells. M2 expression was induced with 3 µg/ml dox for 16 h followed by staining for M2. Scale bar 10 µm. B) IF analysis of M2 localisation in HCT116 EGFP-LC3B TetON-M2 wt, ATG16L1-/- or ATG16L1-/- reconstituted with hATG16L1β cells treated as in A. Scale bar 10 µm.

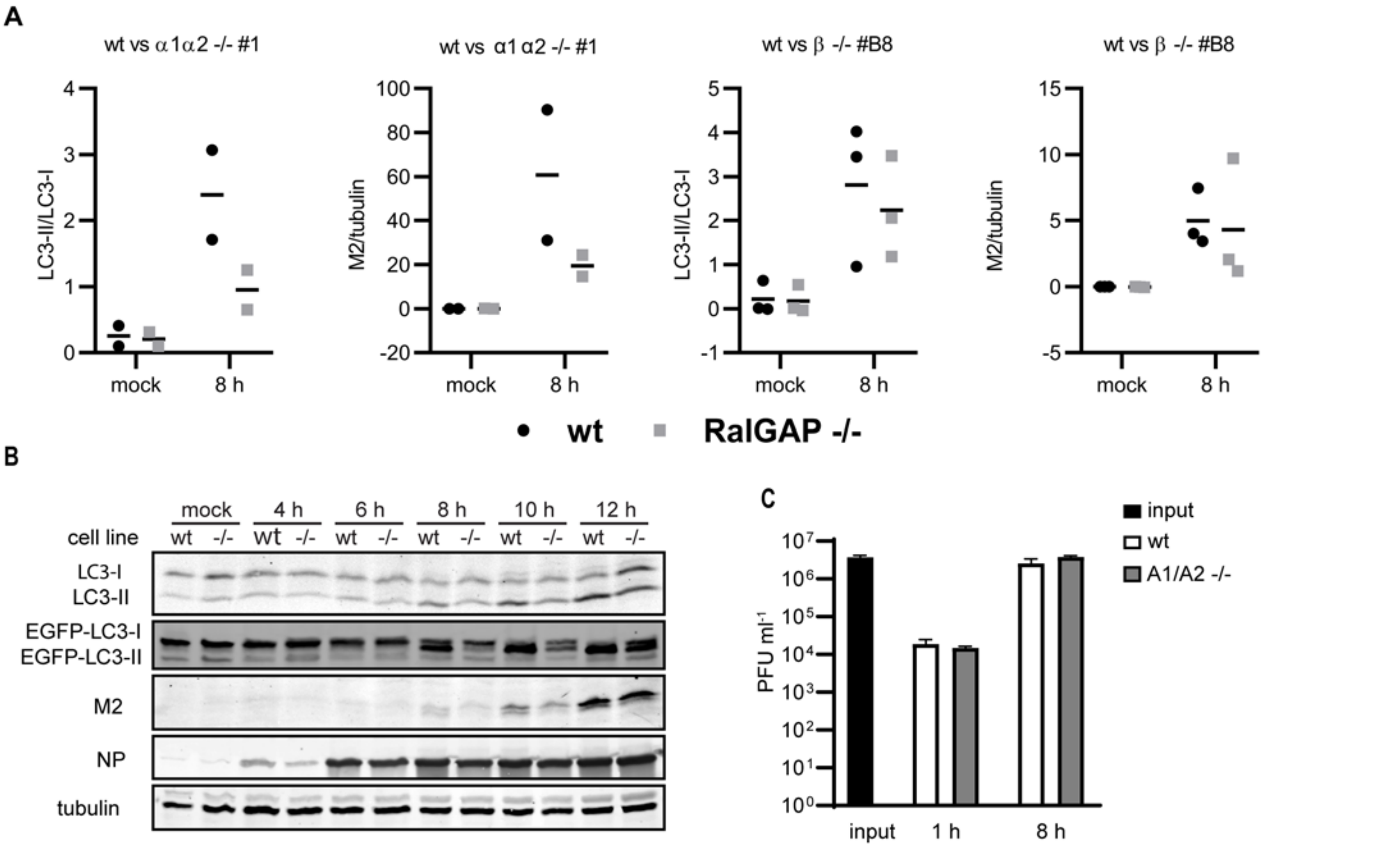

Figure S4, related to figure 3: A) Quantification of LC3-lipidation analysis by western blot of HCT116 EGFP-LC3B TetON-M2 cells depleted for RalGAPA1A2 (panel 1 and 2) or RalGAPB (clone B8) after infection with PR8 at an MOI of 10 PFU per cell for 8 h. Graphs show mean of LC3II/LC3I ratio (panels 1 and 3) and M2 expression normalised to tubulin (panels 2 and 4) from two or three independent experiments. B) LC3-lipidation analysis of HCT116 EGFP-LC3B TetON-M2 wt and cells depleted for RalGAPA1A2 infected with MUd at an MOI of 10 PFU per cell and lysed at the indicated time point p.i.. C) HCT116 EGFP-LC3B TetON-M2 wt or cells depleted for RalGAPA1A2 were infected at an MOI of 10 PFU per cell. Supernatants were harvested at 1 or 8 h p.i. and titres determined by plaque assay.

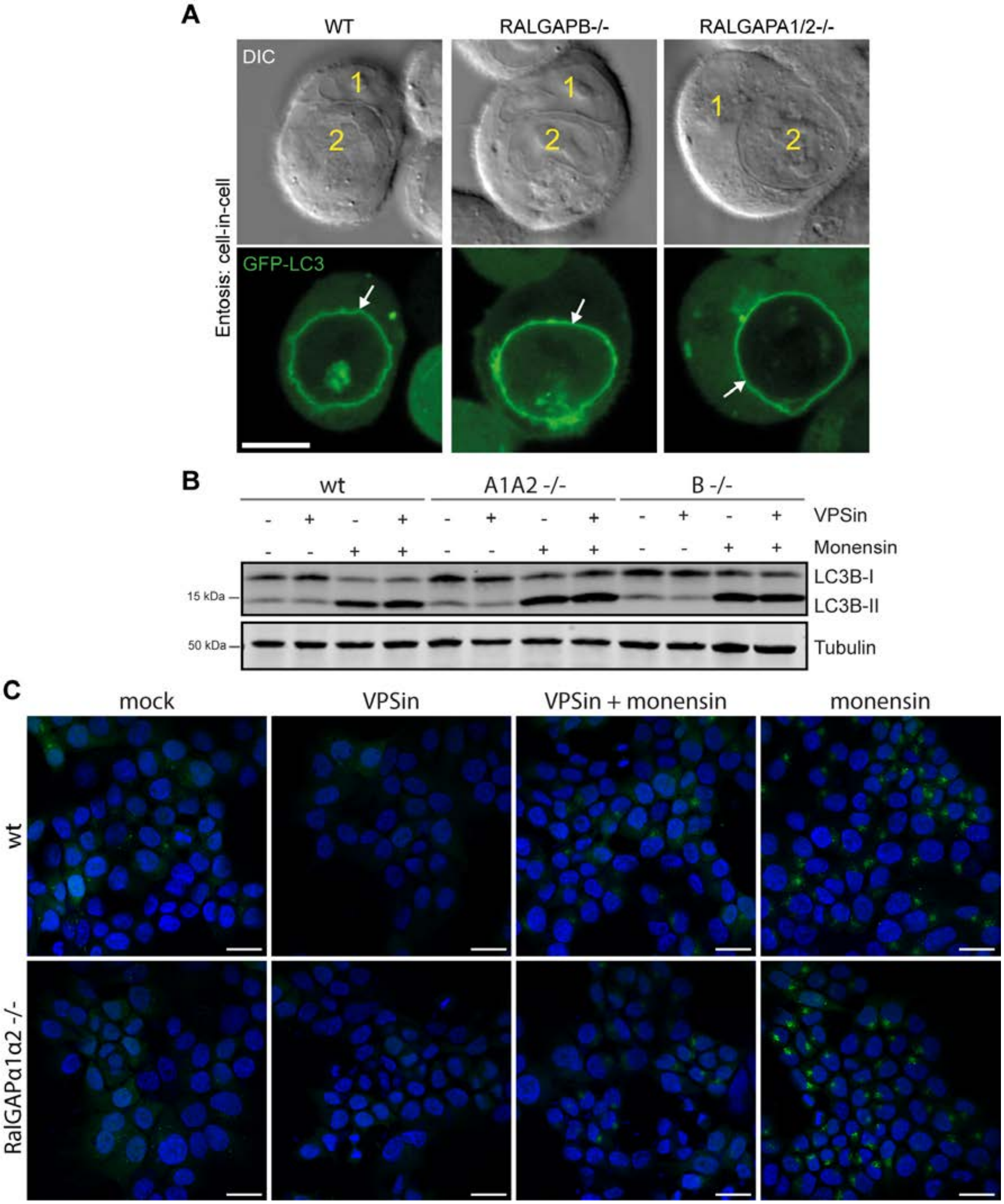

Figure S5, related to figure 3: A) EGFP-LC3B recruitment to entotic vesicles in HCT116 EFGP-LC3B TetON M2 wt, RalGAPα1α2-/- and RalGAPβ-/- cells analysed by fluorescence (top panel) and bright field (bottom panel) live cell microscopy. Scale bar 10 µM. B) LC3 lipidation analysis of HCT116 EFGP-LC3B TetON M2 wt and RalGAPα1α2-/- cells after 30 min treatment with 10 µM monensin. Where indicated cells were pretreated with 1 µM VPSin for 20 min prior to addition of 10 µM monensin to inhibit canonical LC3-lipidation. C) Representative images of IF analysis of HCT116 EFGP-LC3B TetON M2 wt and RalGAPα1α2-/- treated as in B. Scale bar 20 µm.

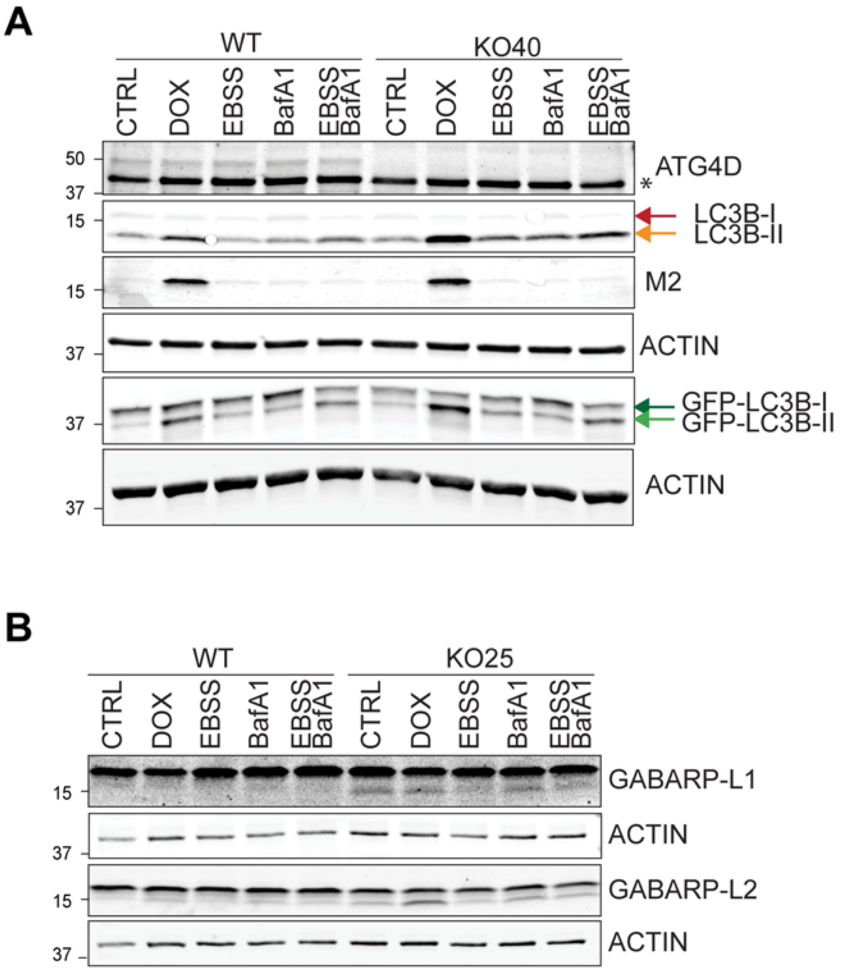

Figure S6, related to figure 4: A) HCT116 EGFP-LC3 TetOn-M2 WT and ATG4D KO clone 40 were treated with 10 µg/ml dox for 8 h, 2 hr with EBSS, 1 hr with 200 nM Bafilomycin A1 and a combination of EBSS together with Bafilomycin A1. arrowhead indicates ATG4D specific band, * background band. B) HCT116 EGFP-LC3 TetOn-M2 WT and ATG4D -/- clone 25 were treated as in B and GABARAP-L1 and GABARAP-L2-lipidation analysed by western blot.
